# A SEMG-Force Estimation Framework Based on a Fast Orthogonal Search Method Coupled with Factorization Algorithms

**DOI:** 10.3390/s18072238

**Published:** 2018-07-11

**Authors:** Xiang Chen, Yuan Yuan, Shuai Cao, Xu Zhang, Xun Chen

**Affiliations:** Department of Electronic Science and Technology, University of Science and Technology of China, Hefei 230027, China; yuan1111@mail.ustc.edu.cn (Y.Y.); caoshuai@ustc.edu.cn (S.C.); xuzhang90@ustc.edu.cn (X.Z.); xunchen@ece.ubc.ca (X.C.)

**Keywords:** high-density SEMG, factorization algorithms, fast orthogonal search, muscle force estimation

## Abstract

A novel framework based on the fast orthogonal search (FOS) method coupled with factorization algorithms was proposed and implemented to realize high-accuracy muscle force estimation via surface electromyogram (SEMG). During static isometric elbow flexion, high-density SEMG (HD-SEMG) signals were recorded from upper arm muscles, and the generated elbow force was measured at the wrist. HD-SEMG signals were decomposed into time-invariant activation patterns and time-varying activation curves using three typical factorization algorithms including principal component analysis (PCA), independent component analysis (ICA), and nonnegative matrix factorization (NMF). The activation signal of the target muscle was obtained by summing the activation curves, and the FOS algorithm was used to create basis functions with activation signals and establish the force estimation model. Static isometric elbow flexion experiments at three target levels were performed on seven male subjects, and the force estimation performances were compared among three typical factorization algorithms as well as a conventional method for extracting the average signal envelope of all HD-SEMG channels (AVG-ENVLP method). The overall root mean square difference (RMSD) values between the measured forces and the estimated forces obtained by different methods were 11.79 ± 4.29% for AVG-ENVLP, 9.74 ± 3.77% for PCA, 9.59 ± 3.81% for ICA, and 9.51 ± 4.82% for NMF. The results demonstrated that, compared to the conventional AVG-ENVLP method, factorization algorithms could substantially improve the performance of force estimation. The FOS method coupled with factorization algorithms provides an effective way to estimate the combined force of multiple muscles and has potential value in the fields of sports biomechanics, gait analysis, prosthesis control strategy, and exoskeleton devices for assisted rehabilitation.

## 1. Introduction

Muscle force estimation refers to the prediction of contraction force produced by a piece of muscle or multiple muscles during human movement. Precise and reliable muscle force estimation is important to many research fields such as sports biomechanics [1,2], gait analysis [3,4,5], prosthesis control strategy [6,7], and exoskeleton devices for assisted rehabilitation [8,9]. Though either invasive [10] or noninvasive [11] methods can predict muscle force, invasive muscle force measurements are not feasible in most clinical settings. Therefore, noninvasive techniques based on musculoskeletal modeling have attracted increasing research attention.

The surface electromyogram (SEMG) is one such noninvasive method to detect muscle activities at the skin surface. It can reflect the compound neuromuscular activity and has been widely used in the field of force estimation. SEMG signals used for muscle force estimation were recorded by one or several separated unipolar or bipolar electrodes in a number of previous related studies [12,13,14,15,16]. According to anthropotomical studies, many skeletal muscles can be subdivided into smaller muscle segments, and there are substantial activation heterogeneities among these segments during muscle contractions [17,18,19,20]. Separated electrodes have limited ability to reflect heterogeneous muscle activities comprehensively, which might limit the force prediction accuracy.

High-density SEMG (HD-SEMG) grids, which consist of tens or even hundreds of electrodes and cover a large portion of target muscles, have shown good performances in some SEMG-based applications including force estimation [21,22,23,24,25]. For example, Staudenmann et al. [25] showed that HD-SEMG signals from the triceps brachii muscle were measured together with the extension elbow force—the effects of the whole grid as well as different bipolar configurations on EMG-based force estimation were analyzed, and the results indicated that the HD-SEMG grid was a powerful tool for force estimation.

In HD-SEMG-based force estimation, researchers found that HD-SEMG signals recorded from target muscles were high dimensional and some of them might be redundant for force estimation. In many previous studies, a common electrode dimension reduction method was to calculate the average rectified signal of all channels or a part of them [23,25,26,27]. However, the simple averaging method can blur the heterogeneity in muscle activation, thus limiting the accuracy of muscle force estimation. Therefore, to get more detailed and useful information for muscle force estimation, it is critical to delve even further into the activation situations of skeletal muscles. Thus, factorization algorithms, such as principal component analysis (PCA), independent component analysis (ICA), and nonnegative matrix factorization (NMF), have been used to analyze the heterogeneity of HD-SEMG signals—they are effective in reducing electrode redundancy and improving the force estimation accuracy [24,25,26,28,29,30].

In one example, Staudenmann et al. compared the performances of several HD-SEMG processing methods in the estimation of elbow extension force and found that PCA coupled with HD-SEMG could markedly reduce the root mean square difference (RMSD) [25]. Huang et al. proposed an isometric muscle force estimation method based on HD-SEMG and NMF algorithm [24]. In their study, the preprocessed SEMG signals were factorized into activation patterns and corresponding time-varying coefficients by NMF algorithm. High weighting channels in the major activation pattern were chosen to extract the input signal of the force estimation model. The results demonstrated that the proposed method could significantly improve the force estimation performance.

Also, a set of physiological and phenomenological models has been proposed to establish the relationship between SEMG and the muscle force. For instance, Bai et al. used a third-order polynomial model to estimate the force exerted by lower limbs with the help of SEMG from the quadriceps femoris and hamstring muscles [31]. Mobasser et al. employed both the multilayer perceptron artificial neural network (ANN) and the radial basis function ANN for hand force estimation during elbow joint movement using upper arm SEMG signals, elbow joint angle, and angular velocity [12]. Johns et al. proposed a method using the fast orthogonal search (FOS) algorithm coupled with an ensemble learning technique to predict isometric contraction force of the right arm and found that the proposed method could improve the estimation performance [21]. The advantage of FOS is that it can rapidly generate the SEMG-force model by selecting suitable basis functions from the candidate pool and balance the modeling time along with the estimation accuracy. Thus, FOS is especially suitable for estimating the combined force of multiple muscles [21,22,32].

To extract more useful muscle activation information from HD-SEMG signals and improve the performance of force prediction, a SEMG-force estimation framework based on the FOS method coupled with factorization algorithms (i.e., PCA, ICA, and NMF) was proposed in this study. More specifically, factorization algorithms were used to decompose HD-SEMG signals from a target muscle into several time-invariant activation patterns and corresponding time-varying activation curves. The activation signal of the target muscle was obtained by summing the activation curves. The FOS method was then utilized to create basis functions constructed by activation signals and establish the force estimation model. The feasibility and superiority of the proposed SEMG-force estimation framework were evaluated under the static isometric elbow flexion task where the generated elbow force was estimated using HD-SEMG signals from the upper arm muscles.

## 2. Materials and Methods

**Figure 1:**
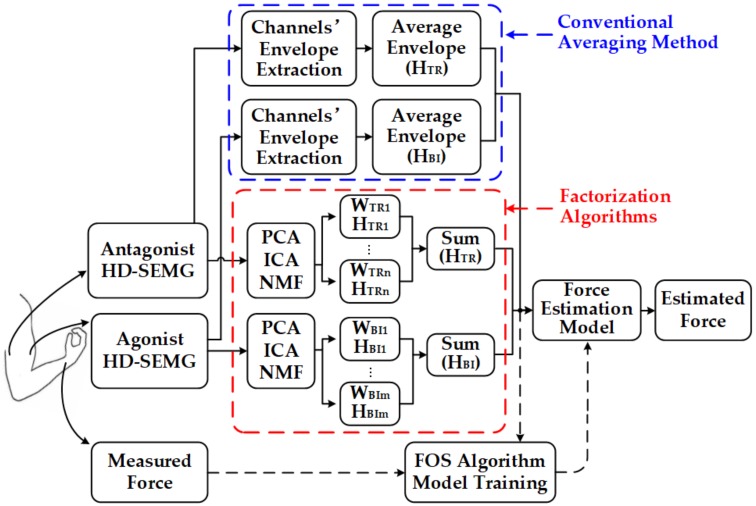
Block diagram of FOS-based HD-SEMG-force estimation framework. (*W*: activation pattern, *H*: activation curve.).

The proposed SEMG-force estimation framework based on factorization algorithms and the FOS method can be summarized into four steps (Figure 1, red dotted frame). First, HD-SEMG signals from the agonist muscle (biceps brachii) and antagonist muscle (triceps brachii) of the upper arm as well as the generated elbow force were recorded during static isometric elbow flexion. Second, HD-SEMG signals were preprocessed and factorized into activation patterns and corresponding activation curves by PCA, ICA, and NMF algorithms. Then activation signals of both agonist muscle (biceps brachii) and antagonist muscle (triceps brachii) were obtained by summing the activation curves of each muscle. These data were input signals for the subsequent FOS method. Finally, the FOS algorithm was utilized to create a basis function pool constructed by activation signals and to establish the force estimation model by choosing suitable functions from the pool.

### 2.1. Data Collection Experiments

In this study, the proposed force estimation framework was evaluated under static isometric elbow flexion task. Seven male subjects aged at 25.4 ± 2.3 (average ± standard deviation), with no known neuromuscular deficits of the right arm, were recruited. Prior to the experiment, all of them signed the informed consent approved by the Ethics Committee on Clinical Research of the First Affiliated Hospital of Anhui Medical University (No. PJ 2017-04-06).

**Figure 2:**
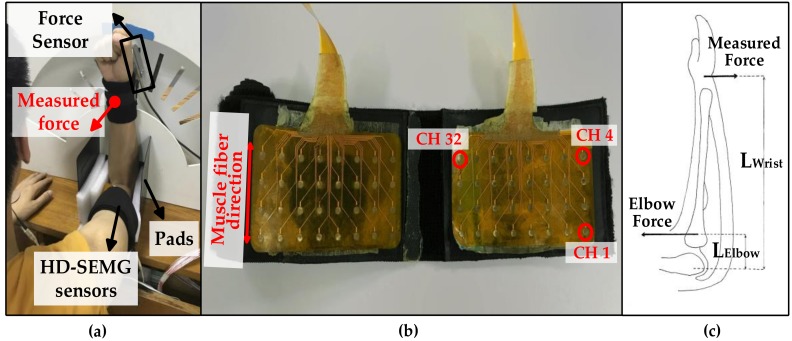
Experimental instrumentation and protocol: (**a**) Illustration of the homemade apparatus and the experimental posture shown by a subject; (**b**) Two high-density electrode grids. (**c**) Free body diagram of the right arm.

The experiments were performed on a homemade apparatus (Figure 2a). A one-dimensional force sensor (LAS-B, Load Cell, Xiamen, China) that could be moved within the groove of the board to adjust the height was used for elbow force measurement. Two high-density electrode grids (Figure 2b) were used for HD-SEMG signals collection of the upper arm muscles—one covered the agonist and the other covered the antagonist. Each grid consisted of 32 metallic electrodes (4 rows × 8 columns), and each electrode had a diameter of 3 mm and a 10 mm center-to-center distance between two adjacent electrodes. All electrode sensors were evenly distributed in a polyimide slice covering a collection surface of 8 cm × 4.6 cm and were differential against the reference electrode pasted on the right elbow. Raw SEMG signals were collected by the homemade acquisition equipment that had a two-stage amplifier with a total gain of 60 dB (about 1400 fold), a built-in band-pass filter (20–500 Hz), and digitalization at a sampling frequency of 1000 Hz using a 16-bit A/D converter (ADS1198). The subject’s skin was shaved and cleaned with alcohol to reduce the skin–electrode impedance, and another reference electrode attached to the system ground was placed at the left hand of subjects; the data acquisition equipment was battery powdered. The SEMG data were monitored on the computer screen in real time. At the beginning of signal processing, very few signals corresponding to missing or bad contacts were checked out and replaced by an adjacent electrode. All data were stored offline for analysis using Matlab R2017a.

During the experiment, subjects were asked to sit upright with the right forearm positioned vertically and the upper arm positioned horizontally. The pads were used to fix the elbows of subjects into the proper position. The force sensor was regulated at the same height as the wrist, and the wrist was tightly tied using an inelastic strap to the board to immobilize the wrist joint decreasing the influence of the wrist force. The subjects were then instructed to keep their left arms and hands in an absolutely relaxed state; the elbow flexion task was performed only on the right upper arm muscles, but this was done as far as possible. Also, their right hand, wrist, and shoulder were all kept static to limit the contributions of forearm and shoulder muscles to the elbow force. The sum of the force moments and the forces acting on the arm could be zero because the isometric elbow flexion task was static—this is obvious in Figure 2c [33]. Consequently, as shown in Equation (1), the measured force at the location of wrist is proportional to the generated force at the elbow:

(1) $$F_{wrist}\times L_{wrist}+F_{elbow}\times L_{elbow}=0$$

Here, $F_{wrist}$ and $F_{elbow}$ represent forces at the wrist and at the elbow, respectively; $L_{wrist}$ and $L_{elbow}$ are the arms of both forces to the elbow joint, respectively.

In the first session, each subject was asked to perform the maximum voluntary contraction (MVC) three times, and the largest value was adopted as the final MVC. In each trial, subjects were asked to generate the measured force increasing linearly from zero to a target value within three seconds while keeping this target value state for another three seconds by performing elbow flexion. The three targeted values were 20%, 40%, and 60% of MVC in the experiment. At each level, the motion task was repeated 7 times. To avoid muscle fatigue, a one-minute rest was enforced between two motion cycles. Before data collection, subjects were asked to practice elbow flexion task using their upper arm muscles until they could successfully track the force profiles.

### 2.2. Extraction of the Input Signals of SEMG-Force Estimation Model

Factorization algorithms including PCA, ICA, and NMF were used to process SEMG signals. In simple terms, factorization algorithms refer to problems where both the sources and the mixing methodology are unknown, and only mixture signals are available for further separation. As shown in Equation (2), factorization algorithm can decompose a matrix $S_{m\times t}$ into two matrixes $W_{m\times n}$ and $H_{n\times t}$ (where n<m). In this study, $S_{m\times t}$ represents the multi-channel SEMG signal matrix (*m* = 32 channels, *t* = 6000 samples), each column of $W_{m\times n}$ represents a time-invariant activation pattern (*n* = 2 or 3 activation patterns) that describes the spatially correlated activations of *m* channels by *m* weighting factors, and each row of $H_{n\times t}$ represents a time-varying activation curve specifying how the corresponding activation pattern is modulated during the static isometric elbow flexion task:

(2) $$S_{m\times t}\approx W_{m\times n}H_{n\times t}$$

More specifically, PCA is an orthogonal feature projection method, which aimed to find orthogonal eigenvalues with greatest variance among the data [34]. PCA projects multivariate data onto a new coordinate system termed as the principal components (PCs). Corresponding eigenvectors and eigenvalues are found after diagonalization of the data’s covariance matrix. The significance of the PCs can be sorted by the eigenvalues, and the variances of the data projected onto the first up to the last component are shown in the decreasing order. This means, in general, that the original data can be well represented by merely the first few PCs. Thus, we are not particularly interested in all PCs but rather extract the first few ones that can already reflect the activation patterns within the target muscle. Here, the original HD-SEMG signals were directly input to PCA. The output eigenvectors were then used to represent the activation patterns within the target muscle, and the PC signals after full-wave rectification and low-pass filtering (window-based finite impulse response filter, Hanning window, 5 Hz, 50th order, similar hereafter) were considered as activation curves for further usage.

ICA is a widely used multivariate data analysis technique that can transform the original vector data into mutually statistically independent components (ICs) [35]. Based on various statistical indexes, ICA determines the independent sources from the observed mixing signals by maximizing statistical independence between data. Since a piece of skeletal muscle can be subdivided into several patterns—and each of them is considered to be independently activated during the muscle contraction process, ICA is an appropriate method to explore the activation of the internal structure within muscles. Here, we used FastICA, which is based on maximum negentropy and was introduced by Hyvärinen [36]. FastICA decomposed the HD-SEMG signals into source signal matrix and corresponding weight coefficient matrix. After full-wave rectification and low-pass filtering, the envelopes of source signals were extracted as activation curves for further processing, and the weight coefficient matrixes represented time-invariant patterns.

NMF was proposed by Lee and Seung [37] and is a relatively late method for blind source separation problems. NMF is an unsupervised learning method for data mining. It can be formulated as a minimization problem with bound constraints. In this algorithm, nonnegativity is the constraint for matrix factorization. The decomposition can be determined by optimizing an error function between the original data matrix and the decomposed ones. NMF uses different cost functions for optimization. It commonly uses two classical matrix decomposition algorithms that minimize the square of the Euclidean distance and minimize the K-L divergence as the cost function. The corresponding multiplicative iteration rules can then not only ensure the non-negative properties of matrixes, but can also converge to the local optimal solution. In this study, due to the nonnegative constraint of NMF, full-wave rectification and low-pass filtering, which were done after factorization in PCA and ICA methods, were proceeded to obtain the envelopes of SEMG signals before factorization. The NMF decomposes the preprocessed HD-SEMG signal matrix into two nonnegative matrices: one represents activation patterns and the other represents activation curves. In this way, the results have more physiological relevance than other matrix factorization algorithms [38].

Considering the anatomical structure of the mainly contained muscles (the biceps brachii has two heads and the triceps brachii muscle has three heads) [39], two activation patterns for the agonist and three activation patterns for the antagonist of the upper arm were finally extracted. The activation curves from the agonist and the antagonist of the upper arm were summed to act as the input signals of the FOS algorithm, which were expressed as $H_{BI}$ and $H_{TR}$.

For comparison, a force estimation framework based on the conventional averaging method (hereafter referred to as AVG-ENVLP method) was also implemented [23,24,25,26,27] (Figure 1, blue dotted frame). In the AVG-ENVLP method, the average signal of all SEMG channel envelopes from each of the two HD-SEMG grids (also expressed as $H_{BI}$ and $H_{TR}$) was calculated and put into FOS-based force model. In addition, the force estimation was also conducted using SEMG signals from only one piece of electrode grids to prove that FOS is especially suitable for the combined force estimation of multiple muscles. All input signals of FOS as well as the measured force were normalized to the maximum value over each movement cycle to facilitate comparison between the measured and estimated forces.

### 2.3. FOS-Based SEMG-Force Estimation Model

The FOS algorithm was first developed by Korenberg [40] and is a nonlinear system identification method that approximates a system output as a weighted sum of basis functions. As shown in Equation (3), y(t) is the measured system output (in this case the measured force at the wrist), $\hat{y}\left(t\right)$ is the estimated system output, $p_{m}\left(t\right)$ are basis functions composed of input signals (in this case the activation signals $H_{BI}$ and $H_{TR}$ extracted from two HD-SEMG grids), $a_{m}$ are the weighting coefficients of basis functions, and e(t) is the estimation error:

(3) $$y\left(t\right)=\hat{y}\left(t\right)+e\left(t\right)=\sum_{m=1}^{M}a_{m}p_{m}\left(t\right)+e\left(t\right)$$

Utilizing the Gram-Schmidt orthogonal method, the candidate basis functions $p_{m}\left(t\right)$ can be converted into a set of mutually orthogonal basis functions $q_{m}\left(t\right)$ (the projection of $q_{r}$ to $p_{m}$ is labeled as $\alpha_{mr}$), and Equation (3) can be rewritten as Equation (4):

(4) $$y\left(t\right)=\sum_{m=1}^{M}g_{m}q_{m}\left(t\right)+e\left(t\right)$$

In this case, it becomes easier to calculate the coefficients of the orthogonal basis functions $g_{m}$ than $a_{m}$. Once $g_{m}$ is acquired, $a_{m}$ can be determined using Equations (5) and (6). A more detailed derivation process of FOS can be found in [40,41]:

(5) $$a_{m}=\sum_{i=m}^{M}g_{i}v_{i}$$

(6) $$v_{i}=\{\begin{array}{c} 1,\;i=\;m\\ -\overset{i-1}{\underset{r=m}{\sum }}\alpha_{ir}v_{r},\;m\;<\;i\;\leq \;M \end{array}$$

Overall, the FOS algorithm searches among a set of candidate functions and selects the function that leads to the highest mean square error (MSE) reduction between the estimated and measured system outputs to generate mathematical model. The model iteratively adds one selected function at a time until some criteria are met. For example, the overall MSE of the FOS estimation model has been small enough; a certain number of functions have been selected for the model; or the remaining candidate functions are unable to significantly reduce the MSE.

Establishing a candidate basis function pool and selecting a function number in the model (the choice of *M*) are two crucial procedures to establish a force estimation model using the FOS method. In related studies [24,25,32,42], the relationship between SEMG signals and the muscle force has been approximately regarded as linear function, polynomial function, exponential function, and sigmoid function. Thus, four subsets of functions including fundamental types, polynomial types (quadratic and cubic), square root functions, and sigmoid functions were considered in this study, as well as an essential bias function. The candidate function pool was built as shown in Table 1 in which $H_{BI}$ and $H_{TR}$ are activation signals extracted from agonist and antagonist, respectively. Moreover, the modeling termination criterion was that the remaining candidate functions could not significantly reduce the estimation error, and the threshold of the RMSD reduction was set to 0.2%.

### 2.4. Evaluation Methods of Force Estimation Performance

To quantify the estimation performance of the trained FOS-based force model, two frequently-used criteria were evaluated: RMSD (which reveals the similarity between the estimated force and the measured force, (7)) and *R*^2^ (which reflects the stability of force estimator performance, (8)):

(7) $$RMSD=\sqrt{\frac{\sum_{i=1}^{T}{\left[\hat{y}\left(i\right)-y\left(i\right)\right]}^{2}}{T}}\times 100\text{\%}$$

(8) $$R^{2}=1-\frac{\sum_{i=1}^{T}{\left[\hat{y}\left(i\right)-y\left(i\right)\right]}^{2}}{\sum_{i=1}^{T}{\left[\bar{y}-y\left(i\right)\right]}^{2}}$$

In Equations (7) and (8), y and $\hat{y}$ represent the measured and estimated force, respectively, $\bar{y}$ is the temporal average of y, and T is the number of samples.

### 2.5. Statistical Analysis

A one-way repeated-measure ANOVA was applied to RMSD and *R*^2^ to better reflect the superiority of the proposed method compared with the conventional AVG-ENVLP method and to examine the significant differences among them. The design included one independent within-subjects factor consisting of four signal processing methods (AVG-ENVLP, PCA, ICA, and NMF). The significance level in the statistical tests was set to *p* < 0.05. All statistical analyses were carried out using SPSS software (version 19.0, IBM, Armonk, NY, USA).

## 3. Results

### 3.1. Representative HD-SEMG Decomposition and Input Signal Extraction Results

**Figure 3:**
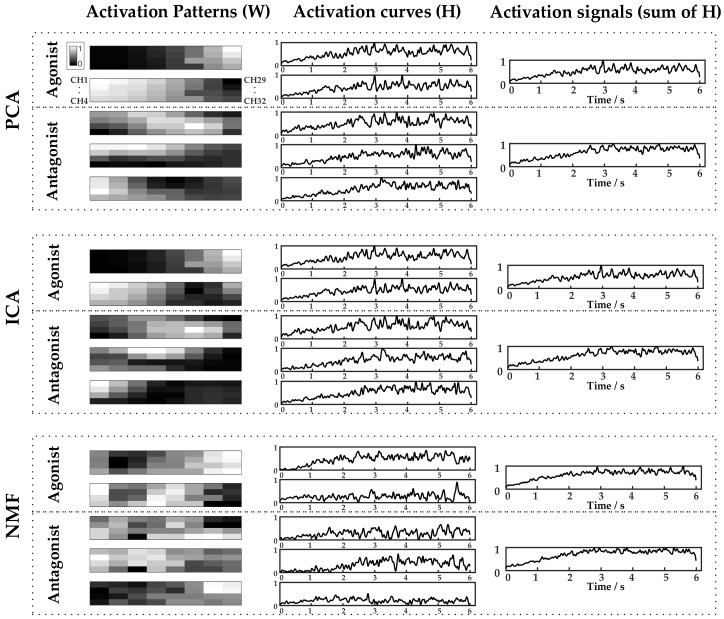
HD-SEMG decomposition and activation signal extraction results obtained via PCA, ICA, and NMF algorithms for Subject 1 during 40% MVC elbow flexion task.

**Figure 4:**
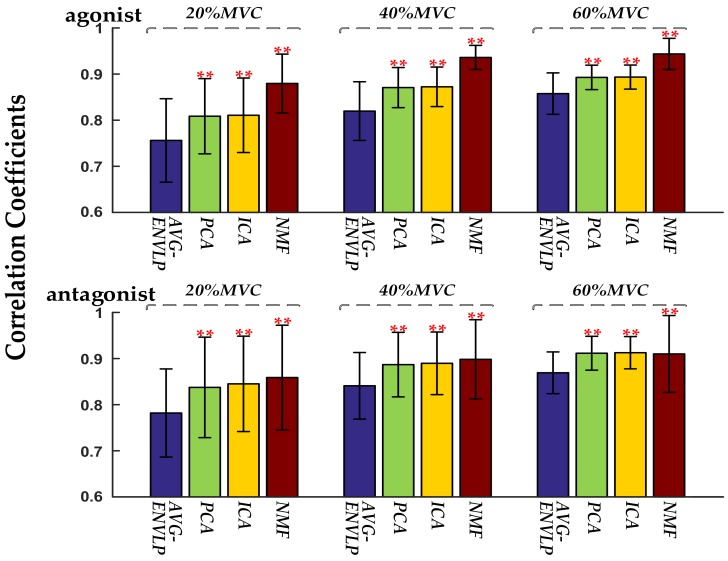
The correlation coefficients between the measured force and the extracted activation signals for all subjects. The significant differences between factorization algorithms and AVG-ENVLP method are marked with ** (*p* < 0.01).

The PCA, ICA, and NMF factorization algorithms were used to extract input signals of the FOS-based force model. Figure 3 gives an example of HD-SEMG decomposition and input signal extraction results obtained via PCA, ICA, and NMF algorithms. Two activation patterns and the corresponding time-varying activation curves were obtained from the agonist, and three activation patterns and the corresponding time-varying activation curves were obtained from the antagonist. The activation patterns were shown as intensity images, and 32 weighting factors from each activation pattern were rearranged into four rows and eight columns and mapped on the form of an electrode grid to get an intensity image. Figure 3 shows the intensity images whereby each activation pattern has its own highlighted area. This indicates that this region is primarily activated. The activation signals (sum of activation curves; input signals for FOS algorithm) are shown as well. The correlation coefficients between the measured force and the activation signals extracted by AVG-ENVLP, PCA, ICA, and NMF for all seven subjects were calculated (Figure 4). The activation signals extracted by factorization algorithms, especially by NMF, are more similar to the measured force signals than that extracted by the conventional AVG-ENVLP method. This provides the possibility for more precise force estimation.

### 3.2. Establishment of FOS-Based SEMG-Force Estimation Model

**Figure 5:**
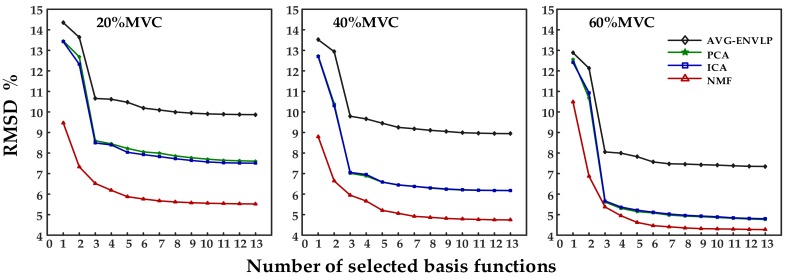
Effect of selected basis function number on RMSD for a representative subject (Subject 1).

**Figure 6:**
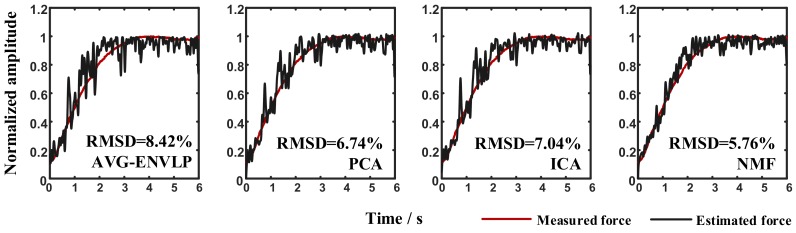
Illustration of the measured force and the estimated force for Subject 1 at 40% MVC task.

Data from Subject 1 were used as an example to determine the number of basis functions and to establish the most suitable force estimation model. Figure 5 shows that when the number of basis functions selected to establish the FOS-based force estimation model moves from 1 to 4, the RMSD value usually decreases over 0.5% by adding one more function to the model. However, the RMSD declines steadily when the function number increases from 4 to 13. In most cases, the force estimation model established with seven basis functions offer good performances. Figure 6 illustrates representative estimated force results when the number of basis functions was set to 7 for Subject 1 at 40% MVC elbow flexion task. Low RMSD values were obtained, especially for NMF algorithm. The statistical analysis of the selected basis functions for all subjects under all conditions is also given in Table 2. Each elbow flexion task was repeated seven times, and the total training number was 588 (7 subjects × 3 force levels × 4 methods × 7 times). The fundamental functions and polynomial functions were chosen more times than square root functions and sigmoid functions, and the selected frequencies of $H_{BI}$, $H_{TR}$ and $H_{BI}\times H_{TR}$ were over 90%. Additionally, functions related to agonist muscles have a higher selected rate than those related to antagonist muscles, e.g., $H_{BI}^{2}$ was selected 491 times while $H_{TR}^{2}$ was selected only 314 times.

### 3.3. SEMG-Force Estimation Results

**Figure 7:**
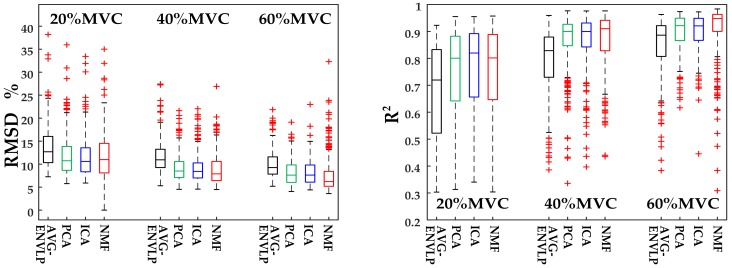
HD-SEMG-based force estimation results for all subjects.

Next, cross-validation force estimation experiments were conducted to verify the proposed force estimation framework. For each subject, one repetition from the seven repetitions was iteratively selected as the training set, and the remaining 6 repetitions were test sets. The overall RMSD and *R*^2^ for all seven subjects are presented in Figure 7. Versus the AVG-ENVLP method, the PCA, ICA, and NMF algorithms can evidently reduce RMSD and improve *R*^2^ values. The RMSD values decrease and R^2^ values increase conversely with force levels rising from 20% MVC to 60% MVC for all subjects.

A one-way repeated-measure ANOVA analysis was performed on RMSD and R^2^ values, and there were significant differences between different methods. Table 3 shows that there are significant differences between the AVG-ENVLP method and the other three factorization algorithms (*p* < 0.0001) for both RMSD and *R*^2^; no such significant difference exists among the three factorization algorithms except for PCA and ICA with *p* < 0.05. Thus, we concluded that the proposed muscle force estimation framework that integrates factorization algorithms could significantly reduce the estimation error compared to the conventional AVG-ENVLP method.

### 3.4. Comparison between Single HD-SEMG Grid and Two HD-SEMG Grids

We also estimated the measured force using signals from only one piece of HD-SEMG grid via the NMF + FOS framework. This better reflects the performance of the proposed framework and proves that FOS is especially suitable for estimating the combined force of multiple muscles. Here, the number of FOS input signals changed from 2 to 1, and the number of basis functions was reduced to 5 (including H, $H^{2}$, $H^{3}$, $\sqrt{H}$, and sigm(H)).

**Figure 8:**
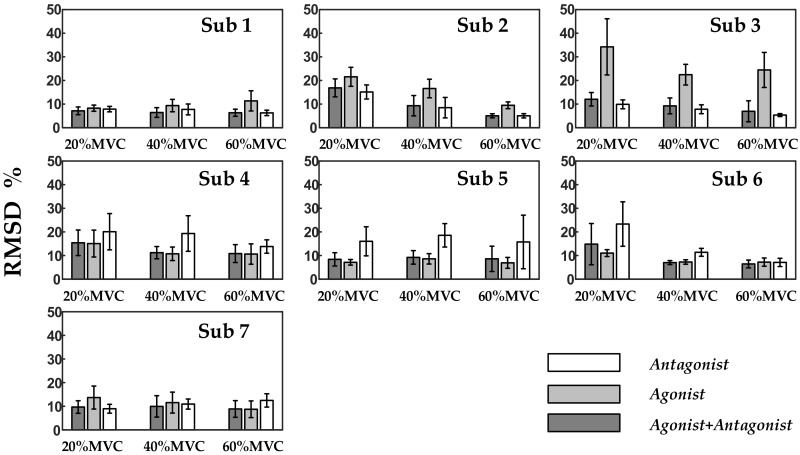
Force estimation results corresponding to the agonist, the antagonist, and both of them, respectively.

Figure 8 illustrates the force estimation results obtained using a HD-SEMG grid corresponding to the agonist, the antagonist, and both for seven subjects. The performance can vary markedly across different individuals when SEMG signals from only one piece of grid were used to estimate the measured force. For subjects 4, 5, and 6, the force estimation was more accurate when signals from the agonist muscles were used. For subjects 1, 2, and 3, the situation was the opposite. In general, relatively small RMSD values were obtained when the force was estimated by combining the agonist muscle and the antagonist muscle. Table 4 lists the average and standard deviation for seven subjects—the RMSD values are lower and R^2^ values are higher when using SEMG signals from two muscles to estimate the measured force.

## 4. Discussion

Previous studies have demonstrated that SEMG signals can offer a comparatively accurate estimation of relevant force. In most HD-SEMG based force estimation studies [21,23,24,25], only a few signal channels were selected for force estimation. Such approaches usually used some representative channels and averaged those signals to obtain one activation curve. The framework proposed here decomposed HD-SEMG signals into distinguishable activation patterns within muscles via factorization algorithms and then used the extracted activation signals to estimate muscle force via the FOS algorithm. The results showed that the FOS algorithm with input signals ameliorated by factorization algorithms could offer high SEMG-force estimation accuracy and stability (RMSD between the normalized estimated and measured forces: 11.79 ± 4.29% for AVG-ENVLP, 9.74 ± 3.77% for PCA, 9.59 ± 3.81% for ICA, and 9.51 ± 4.82% for NMF). Of the three factorization algorithms, NMF provided the best results for all subjects—especially under high force levels. Moreover, the force estimation performance was improved using SEMG signals from both agonist and antagonist muscles compared to using SEMG from only the agonist or the antagonist.

The present study deeply explored the activation of target muscles via factorization algorithms such as PCA, ICA, and NMF. The factorization algorithms could decompose HD-SEMG signals into different activation patterns and corresponding activation curves (Figure 3). The activation patterns show that different activation regions of the upper arm muscles (biceps brachii and triceps brachii) could be extracted during the static isometric elbow flexion task. The active curves specify how corresponding activation patterns are modulated during the movement and contain detailed muscle activation information. The sums of the active curves extracted using factorization algorithms are more similar to the measured force signal than the activation signals extracted using the conventional AVG-ENVLP method (Figure 4). This phenomenon lays the foundation for improving the accuracy of muscle force estimation. Figure 7 and Table 3 show that the force models based on factorization algorithms, especially NMF, offered substantial improvements in RMSD and *R*^2^ versus AVG-ENVLP.

In addition, because it contained multitudinous nonlinear basis functions, FOS offered better estimation performance than simple linear model used in previous studies [43]. Taking the representative data of Subject 1 at 40% MVC as an example, average RMSDs were 10.25% for AVG-ENVLP, 7.03% for PCA, 6.90% for ICA, and 6.22% for NMF when FOS model was adopted, however, 13.62% for AVG-ENVLP, 10.37% for PCA, 10.15% for ICA, 7.29% for NMF when simple linear model was used. What has to be pointed out is, FOS caused longer training time than the linear model. Besides, the advantages of FOS in estimating the combined force of multiple muscles were detailed in Figure 8 and Table 4. When signals from only one piece of SEMG grid were considered, the number of candidate functions for force estimation model was reduced, and the force estimation performances were unstable among individuals. However, when SEMG signals from both of the HD-SEMG grids were considered simultaneously, more basis functions could be built to establish the force estimation model. This improved the quality and stability of force estimation. In previous studies related to the FOS algorithm, even more muscles were considered for force estimation. For instance, Mobasser et al. [32] showed that the elbow-induced wrist force was estimated via EMG signals from biceps, triceps, and brachioradialis muscles along with the elbow joint angle and angular velocity. They obtained a 10% relative mean square error percentage (MSE%) from a representative subject with 10 basis functions in the FOS model. Here, the performances of the proposed force estimation method for all subjects at 60% MVC were about 5% according to the evaluation standard MSE. Consequently, we believe that the FOS method coupled with factorization algorithms is an effective way to estimate the combined force of multiple muscles.

In summary, this study validated the feasibility of a novel muscle force estimation framework based on factorization algorithms and the FOS algorithm. Only the SEMG signals from the upper arm muscles were collected to estimate the generated elbow force. While the subjects were asked to avoid activation of forearm muscles during elbow flexion task, forearm muscles inevitably have some contribution to the force measured at the wrist. This is the source of some errors between the estimated and measured forces. Consequently, contributions from the forearm muscles including brachioradialis, flexor carpi radialis, and extensor digitorum should be considered in future work. In addition, apart from the elbow-induced force at the wrist, other combined forces of multiple muscles could be studied to verify the feasibility of the proposed force estimation framework. Further research will also evaluate dynamic contraction tasks.

## 5. Conclusions

A HD-SEMG-force estimation framework based on FOS and factorization algorithms was proposed and implemented, and its feasibility and superiority were verified under static isometric elbow flexion task. The results demonstrated that factorization algorithms could extract more detailed muscle activation information at a deeper level than the conventional method. Coupled with factorization algorithms, fast orthogonal search method provided an effective way to realize the combined force estimation of multiple muscles. The framework proposed here can be employed in many significant applications, including sports biomechanics, gait analysis, myoelectric prosthesis, and control of exoskeleton devices.

## Figures and Tables

**Table 1:** Candidate basis functions for FOS modeling.

Subset Type	Function Form
Fundamental functions	$H_{BI}H_{TR}H_{BI}\times H_{TR}$
Polynomial functions	$H_{BI}^{2}H_{TR}^{2}H_{BI}^{3}H_{TR}^{3}$
Square root functions	$\sqrt{H_{BI}}\sqrt{H_{TR}}\sqrt{H_{BI}\times H_{TR}}$
Sigmoid functions	$sigm\left(H_{BI}\right)sigm\left(H_{TR}\right)sigm\left(H_{BI}\right)\times sigm\left(H_{TR}\right)$

**Table 2:** Times and frequency of basis function selection for all subjects.

Fundamental Functions			Square Root Functions		
$H_{BI}$	584	99.32%	$\sqrt{H_{BI}}$	285	48.47%
$H_{TR}$	580	98.64%	$\sqrt{H_{TR}}$	146	24.83%
$H_{BI}\times H_{TR}$	561	95.41%	$\sqrt{H_{BI}\times H_{TR}}$	96	16.33%
**Polynomial Functions**			**Sigmoid Functions**		
$H_{BI}^{2}$	491	83.50%	$sigm\left(H_{BI}\right)$	173	29.42%
$H_{TR}^{2}$	314	53.40%	$sigm\left(H_{TR}\right)$	97	16.50%
$H_{BI}^{3}$	464	78.91%	$sigm\left(H_{BI}\right)\times sigm\left(H_{TR}\right)$	47	7.99%
$H_{TR}^{3}$	278	47.28%			

**Table 3:** The *p* values of the paired tests in ANOVA.

Method	AVG – PCA	AVG − ICA	AVG − NMF	PCA − ICA	PCA − NMF	ICA − NMF
**RMSD**	3.13 × 10^−7^	2.20 × 10^−8^	1.80 × 10^−7^	0.023	0.070	0.242
**R^2^**	6.50 × 10^−5^	1.84 × 10^−5^	5.57 × 10^−6^	0.005	0.062	0.302

**Table 4:** The force estimation RMSD and *R*^2^ (average ± standard deviation) for all subjects. (AG: agonist muscle, ANT: antagonist muscle).

	20% MVC			40% MVC			60% MVC		
	AG + ANT	AG	ANT	AG + ANT	AG	ANT	AG + ANT	AG	ANT
**RMSD**	12.05 ± 5.71	15.85 ± 10.34	14.47 ± 7.63	8.91 ± 3.49	12.37 ± 5.93	12.03 ± 6.10	7.58 ± 3.82	11.25 ± 6.89	9.39 ± 6.18
**R^2^**	0.73 ± 0.22	0.60 ± 0.32	0.63 ± 0.31	0.87 ± 0.12	0.73 ± 0.27	0.75 ± 0.24	0.90 ± 0.13	0.78 ± 0.27	0.85 ± 0.20
